# Designing tech-ready recreational sport systems: a conceptual analysis of unified and modular architectures

**DOI:** 10.3389/fspor.2026.1767256

**Published:** 2026-06-24

**Authors:** Jun Woo Kwon

**Affiliations:** Department of Physical Education, Sports Technology Laboratory, Seoul National University, Seoul, Republic of Korea

**Keywords:** modular system, participation models, recreational sport, staged development, technology adoption

## Abstract

Recreational sport represents the largest and most diverse segment of global sport participation, yet sport science and coaching research remains largely centered on elite performance contexts and technology-driven interventions. As a result, many recreational sport programs lack coherent system-level structures that support long-term participation, adaptability across skill levels, and scalable innovation. This conceptual analysis synthesizes literature on recreational sport systems, participation models, staged development frameworks, and technology adoption theory to advance a system-oriented perspective on recreational sport design. Drawing on established participation frameworks—including recruitment–retention–transition models, the Developmental Model of Sport Participation, and Long-Term Athlete Development—recreational sport is conceptualized as a dynamic system rather than a collection of isolated instructional practices. Participation is framed as a staged and modular process shaped by organizational capacity, program architecture, and participant diversity. The analysis further integrates theories of technology adoption and diffusion to examine how field-based sport technologies can be embedded within recreational sport systems. Rather than treating technology as a standalone solution, it is positioned as a modular system component whose effectiveness depends on alignment with program structure, participant readiness, and pedagogical intent. By integrating participation theory, system architecture, and technology readiness, this paper proposes a conceptual and hypothesis-generating framework for designing recreational sport systems that are inclusive, adaptable, and resilient, and for evaluating emerging sport technologies within broader participation ecosystems. The framework is exploratory rather than empirically validated, and its proposed relationships require future longitudinal testing using observable indicators of participation continuity, sustained adoption, dropout, participant readiness, and organizational capacity.

## 1 Introduction

Recreational sport constitutes the broadest and most heterogeneous domain of sport participation globally, encompassing individuals with wide variation in age, skill level, motivation, and social context. In contrast to elite sport—where performance optimization and competitive outcomes dominate—recreational sport systems must respond to a plurality of participant goals, including enjoyment, health maintenance, skill development, social interaction, and sustained engagement over time. Despite this breadth, a substantial proportion of sport science and coaching research continues to privilege elite-performance paradigms, laboratory-controlled methods, and technology-centered interventions that often fail to reflect the operational realities of community-based and recreational sport environments.

This imbalance has contributed to a persistent conceptual disconnect within the literature. Recreational sport is frequently approached as a simplified extension of elite sport rather than as a domain governed by its own structural, pedagogical, and organizational principles. Participation is commonly framed in terms of individual motivation or discrete coaching practices, while the system-level architecture that shapes recruitment, retention, transition, and disengagement remains insufficiently theorized (1, 2). Consequently, many recreational sport programs encounter recurring difficulties in maintaining participation, responding to participant diversity, and incorporating innovation in ways that are scalable and contextually appropriate.

### 1.1 Recreational sport as a systemic problem

Foundational contributions in sport management have emphasized that participation should not be understood as a fixed outcome but as an evolving process embedded within organizational and programmatic structures. Green's recruitment–retention–transition framework conceptualizes participation as movement through interconnected program pathways, shaped primarily by system design rather than individual preference alone. Complementary research in community sport further identifies organizational capacity, governance arrangements, and resource alignment as central determinants of program sustainability (2). Together, these perspectives suggest that recreational sport is best conceptualized as a system that coordinates participants, programs, coaches, technologies, and institutions across time.

Nevertheless, dominant participation models remain fragmented across disciplinary boundaries. Economic and behavioral approaches tend to emphasize individual cost–benefit calculations, time constraints, or health-related incentives, whereas psychosocial models focus on attitudes, identity formation, and motivational regulation (3). While each perspective offers valuable insight, they rarely converge into a coherent system-level framework capable of guiding program design or supporting long-term participation strategies. As a result, recreational sport systems are often assembled in an *ad hoc* manner, with limited alignment across participation stages or levels of ability.

### 1.2 Staged participation and program architecture

Developmental frameworks in sport offer a partial response to this fragmentation by conceptualizing participation as a staged process. Models such as the Developmental Model of Sport Participation and Long-Term Athlete Development describe trajectories that extend from early sampling and play toward specialization, sustained engagement, or lifelong involvement (4–6). Although these frameworks are most frequently applied within talent development contexts, their core logic—progression, transition, and differentiation—has direct relevance for the design of recreational sport systems.

In practice, however, staged participation models are rarely implemented at the level of system architecture in recreational settings. Programs often rely either on unified designs that impose standardized structures across all participants or on loosely connected modular offerings that lack coordination and continuity. Without an explicit architectural perspective, it becomes difficult to align coaching practices, learning objectives, and participant experiences across stages of engagement. As a result, recreational sport systems frequently struggle to facilitate smooth transitions, support re-entry following dropout, or adapt effectively to changing participant needs across the lifespan (7, 8).

### 1.3 Technology, innovation, and scalability in recreational sport

Alongside these structural challenges, recreational sport has been characterized by the rapid introduction of digital technologies, including wearable devices, mobile applications, and field-based performance analysis tools. Such technologies are often promoted as mechanisms for enhancing engagement, learning, and feedback. However, accumulating evidence indicates that many sport technologies fail to achieve sustained adoption outside elite or highly controlled contexts. Factors such as user acceptance, perceived usefulness, ease of use, and organizational readiness play a decisive role in determining whether technologies become meaningfully integrated into everyday practice (9–11).

From a system-level standpoint, technology should therefore not be treated as an isolated intervention but as a component whose effectiveness depends on alignment with program design, participant readiness, and pedagogical intent. When this alignment is absent, technological innovations risk adding complexity, exacerbating inequalities in access, or remaining confined to experimental or elite settings. These challenges are particularly acute in recreational sport, where inclusivity, scalability, and limited resources are defining constraints (12).

### 1.4 Purpose and contribution of the present study

Within this context, the purpose of the present paper is to advance a conceptual, system-oriented framework for recreational sport design that integrates participation theory, staged development models, and principles of technology readiness. Rather than examining a specific sport, technological tool, or intervention, this conceptual analysis reframes recreational sport as an adaptive system composed of modular elements—program stages, coaching practices, and technological components—that must be coherently aligned to support sustained participation. As a conceptual analysis, the framework is intended to be exploratory and hypothesis-generating rather than empirically validated.

By synthesizing literature from sport management, motor development, participation modeling, and technology adoption, this paper makes three contributions. First, it clarifies the distinction between unified and modular program architectures in recreational sport systems. Second, it situates staged participation models within a broader system design perspective, emphasizing the importance of transitions, adaptability, and long-term sustainability. Third, it provides a conceptual basis for evaluating emerging sport technologies not as standalone solutions, but as system-dependent tools whose value is realized only when embedded within appropriate program structures.

Through this lens, the analysis shifts attention away from isolated coaching methods or technological artifacts and toward the architecture of recreational sport systems themselves. This perspective offers researchers, practitioners, and policymakers a theoretical foundation for designing recreational sport programs that are inclusive, scalable, and resilient, while remaining responsive to the diverse motivations and capacities of participants.

### 1.5 Novel contribution and propositions

Although existing participation frameworks have clarified how sport engagement evolves across developmental stages, relatively little attention has been given to the architectural design of recreational sport systems that coordinate these stages. The present study extends prior research by conceptualizing recreational sport participation as a system architecture composed of interconnected modules rather than as a linear developmental pathway.

In this framework, modularity functions as a system design principle that enables multiple participation trajectories, while technology is positioned as a modular enabler that can support specific components of the participation system without defining the system itself.

Based on this conceptual synthesis, the following propositions may guide future empirical research:

Conceptual Proposition 1: Modular recreational sport architectures may be associated with greater participation continuity than unified program structures, insofar as they provide multiple entry, transition, and re-entry pathways. This proposition requires empirical validation through longitudinal research.
Conceptual Proposition 2: Technologies embedded as optional modules within participation systems may support sustained adoption more effectively than technologies imposed as mandatory system components, particularly when they align with participant readiness, organizational capacity, and pedagogical objectives.
Conceptual Proposition 3: Recreational sport systems that explicitly design transition and re-entry pathways may reduce the likelihood that temporary withdrawal becomes long-term dropout. This proposition requires future empirical testing using longitudinal participation data.

## 2 Recreational sport as a system

Recreational sport is commonly described through the lenses of specific activities, programs, or participant characteristics. While such descriptions are useful at a descriptive level, they often obscure the structural conditions that shape participation over time. When recreational sport is reduced to a collection of instructional practices or leisure offerings, persistent challenges—such as participant dropout, unequal access, limited adaptability, and weak scalability—tend to be interpreted as isolated issues rather than as outcomes of underlying system design. A system-oriented perspective provides a more integrative framework for understanding how recreational sport participation is produced, organized, and sustained.

### 2.1 From individual participation to system-level design

Early theoretical approaches to sport participation focused predominantly on the individual. Economic and behavioral models framed participation as a function of time allocation, perceived benefits, and opportunity costs, while psychosocial perspectives emphasized attitudes, identity formation, and motivational regulation (3). These approaches offer important explanations for why individuals choose to initiate or discontinue participation, yet they provide limited direction for how sport programs should be structured to support diverse and evolving participation pathways.

In response to this limitation, sport management research has increasingly emphasized the role of organizational systems in shaping participation outcomes. Green's recruitment–retention–transition framework represents a significant shift away from binary participation models by conceptualizing engagement as movement through interconnected sport programs, structured by design decisions rather than individual motivation alone (1). Within this framework, dropout and sustained engagement are understood as consequences of how programs are organized, sequenced, and linked across time.

This system-level perspective is particularly salient in recreational sport contexts, where participant goals are diverse and often change over time. Unlike elite sport systems—typically characterized by hierarchical performance structures and formal selection mechanisms—recreational sport systems must accommodate multiple modes of entry, exit, re-entry, and lateral movement across programs. In the absence of an explicit system architecture, recreational sport provision is prone to fragmentation, reactive decision-making, and inequitable access.

### 2.2 Organizational capacity and community sport systems

Recreational and community sport programs operate within organizational environments that both constrain and enable participation. Research on organizational capacity demonstrates that program sustainability depends not only on participant demand, but also on governance arrangements, human resources, financial stability, and strategic coherence (2). These organizational conditions directly influence the capacity of sport providers to design coherent participation pathways, respond to changing participant needs, and integrate innovation in a responsible manner.

From a system standpoint, recreational sport organizations function as interconnected nodes within broader participation ecosystems that include schools, clubs, municipal agencies, and informal providers. The effectiveness of these ecosystems depends less on isolated program quality than on coordination across providers. When programs are developed without consideration of their position within the wider system, participants are more likely to experience discontinuities—such as abrupt transitions between age groups, skill levels, or program formats—that undermine long-term engagement.

Comparative studies of sport systems further highlight that participation outcomes are strongly shaped by system architecture rather than by isolated program features. Although this body of literature has primarily examined elite sport, it offers valuable conceptual insights into how policy frameworks, institutional structures, and organizational alignment influence participation more broadly (13). These insights suggest that recreational sport systems, like elite systems, require intentional design to balance inclusivity, progression, and sustainability.

### 2.3 Recreational sport as a dynamic, adaptive system

Conceptualizing recreational sport as a system also requires acknowledging its dynamic and adaptive nature. Participation patterns evolve across the lifespan, shaped by developmental processes, social contexts, and changing personal circumstances. Research in motor development demonstrates that coordination, control, and learning capacities change substantially from childhood through adulthood, underscoring the need for adaptable program designs rather than fixed instructional models (8). Similarly, work on expertise and talent development emphasizes that sport engagement follows non-linear trajectories, characterized by phases of exploration, consolidation, and transformation (7).

Complex systems perspectives reinforce this understanding by highlighting that sport participation emerges from interactions among multiple components rather than from linear cause–effect relationships (14). Within recreational sport systems, seemingly minor design decisions—such as entry criteria, feedback structures, or scheduling practices—can generate disproportionate effects on participation trajectories. A system-oriented approach therefore directs attention toward relationships, feedback loops, and adaptability rather than toward isolated interventions or singular outcomes.

### 2.4 Implications for system architecture

Viewing recreational sport through a system lens shifts analytical focus from the question of “what activities are offered” to the more fundamental issue of “how participation is structured.” This shift foregrounds architectural questions: How are programs connected across participation stages? How are transitions managed over time? In what ways does the system accommodate diversity in ability, motivation, and commitment? Addressing these questions requires moving beyond unified program models that impose standardized structures, toward modular architectures that allow flexibility while maintaining overall coherence.

Crucially, this system perspective also establishes the conditions for understanding innovation in recreational sport. Technologies, pedagogical approaches, and program formats do not function independently; their impact is contingent on how they are embedded within the broader system. In the absence of a clearly articulated system architecture, innovations are likely to remain peripheral, unevenly distributed, or unsustainable.

## 3 Staged participation and program architecture

Interpreting recreational sport as a system requires explicit consideration of how participation develops over time. Participation is not a fixed condition but a dynamic process that may involve entry, progression, transition, stagnation, re-engagement, and eventual exit. Staged participation models offer a conceptual framework for capturing this temporal dimension of engagement. However, their implications for the architectural design of recreational sport systems have received comparatively limited attention. This section examines staged participation frameworks as a form of system logic and contrasts unified and modular program architectures as alternative approaches to structuring recreational sport participation.

### 3.1 Staged participation as a system logic

Models of sport participation and development have consistently emphasized that engagement unfolds through identifiable stages rather than as a uniform experience. The Developmental Model of Sport Participation (DMSP) proposes that sport engagement evolves through stages such as sampling, specialization, investment, and recreational participation, reflecting changing motivations and levels of commitment across the lifespan (5, 6). Similarly, Long-Term Athlete Development (LTAD) frameworks describe stage-based pathways aligned with biological maturation, skill development, and psychosocial growth (4).

Although these models were initially developed within talent development and high-performance contexts, their central contribution lies in conceptualizing participation as a staged process. From a system perspective, stages can be understood as functional states within a participation architecture. Each state is associated with distinct learning priorities, motivational orientations, physical capacities, and support requirements. Recreational sport systems that ignore these distinctions often rely on uniform program structures that fail to align with participant needs, thereby increasing the risk of disengagement or dropout.

Staged participation frameworks also align with foundational theories of motor learning and development, which demonstrate that learning processes, coordination strategies, and adaptability change across the lifespan (15, 16). These perspectives challenge assumptions of linear progression and stable participant characteristics, reinforcing the need for program designs capable of accommodating variability and developmental change.

### 3.2 Unified program design in recreational sport

Unified program design refers to system architectures in which a single, standardized structure is applied to all participants regardless of stage, ability, or motivation. Such designs often prioritize consistency, administrative efficiency, and scalability through uniform rules, curricula, or coaching approaches. In recreational sport contexts, unified designs are frequently observed in school-based programs, municipal offerings, and entry-level club structures where resource constraints and organizational simplicity are salient concerns.

While unified designs may support initial access and ease of administration, they often struggle to accommodate heterogeneity in participant goals and developmental trajectories. Research in motor learning indicates that effective learning environments require practice conditions, feedback, and task constraints to be aligned with learner readiness and intent (16, 17). When unified programs impose identical demands across participants, they risk under-stimulating some individuals while overwhelming others.

Unified architectures also tend to obscure transition points within participation pathways. Participants who outgrow or diverge from the standardized structure may have limited options for progression or adaptation, leading to system exit rather than transition. This limitation is particularly problematic for recreational sport systems that aim to support lifelong participation rather than channel participants toward narrow performance-oriented outcomes.

### 3.3 Modular program architecture and adaptability

In contrast, modular program architecture conceptualizes recreational sport systems as assemblages of interconnected yet semi-autonomous components. Modules may vary according to skill level, age group, motivational focus, instructional format, or degree of technological integration, while remaining connected through shared principles and defined transition mechanisms. This architectural approach aligns closely with staged participation models, as each module can be tailored to the functional requirements of a particular participation stage.

From a system design perspective, modular architectures offer several advantages. They enhance flexibility by allowing participants to enter, exit, and re-enter the system at multiple points without undermining overall coherence. They also support adaptability, as individual modules can evolve in response to shifting participant demographics, resource availability, or pedagogical innovation. Importantly, modularity promotes inclusivity by legitimizing multiple participation pathways rather than enforcing a single normative trajectory.

The benefits of modular design are further supported by complex systems research in sport, which emphasizes that adaptive behavior emerges from interactions among system components rather than from centralized, rigid control (14). Within modular recreational sport systems, feedback loops between participants, coaches, and programs can be used to refine individual modules without destabilizing the broader system.

### 3.4 Participation continuity, transition, and re-engagement

From the conceptual perspective advanced in this paper, a potential advantage of modular program architecture is its capacity to support participation continuity across the lifespan. Empirical and conceptual work on sport participation indicates that disengagement is often temporary and context-dependent rather than permanent (1). Systems that lack explicit re-entry pathways risk converting short-term withdrawal into long-term dropout.

Psychological models such as the Psychological Continuum Model conceptualize participation as movement through stages of awareness, attraction, attachment, and allegiance, emphasizing that psychological engagement fluctuates over time (18). From a system perspective, these fluctuations necessitate architectural features that allow participants to realign their identities and motivations with available program structures. Modular designs facilitate this realignment by offering alternative entry points and participation formats without requiring complete system exit.

Socioecological approaches to physical activity further highlight that participation is shaped by interactions among individual, social, organizational, and environmental factors (19). Recreational sport systems that acknowledge these multi-level influences are better equipped to design modules that respond to contextual barriers and facilitators, including time constraints, social support, and access to facilities. In this way, modular architecture supports both developmental staging and contextual responsiveness.

### 3.5 Implications for recreational sport system design

The contrast between unified and modular program architectures carries important implications for the design of recreational sport systems. Unified designs emphasize simplicity and control but often do so at the expense of adaptability and long-term engagement. Modular designs introduce greater organizational complexity, yet they align more closely with staged participation models, motor learning principles, and the realities of heterogeneous recreational sport populations.

Importantly, modularity should not be equated with fragmentation. Effective recreational sport systems require coordination mechanisms—such as shared values, transparent transition criteria, and communication structures—that preserve coherence across modules. Program design frameworks and recreational sport programming literature consistently emphasize the need to align leadership, pedagogy, and operational practices to achieve this coherence.

By embedding staged participation models within a broader system architecture perspective, this section underscores that participation theory alone is insufficient to address persistent challenges in recreational sport. The central issue is not only how participation unfolds, but how systems are intentionally designed to support, adapt to, and sustain participation across stages and contexts.

To further clarify the operational differences between unified and modular participation architectures, Table 1 summarizes key design dimensions relevant to recreational sport systems.

**Table 1:** Conceptual comparison of unified and modular recreational sport system architectures and potential operational indicators.

Dimension	Unified architecture	Modular architecture	Potential operational indicators
Program structure	Single standardized participation pathway	Multiple interconnected participation modules	Number of distinct program modules; degree of formal linkage between modules
Entry points	Limited entry points	Multiple entry points	Number of formal entry routes; proportion of participants entering through non-initial modules
Participant progression	Predominantly linear progression	Flexible and non-linear progression	Frequency of module switching; lateral movement between modules; non-linear progression records
Governance	Centralized program governance	Distributed coordination across modules	Number of coordinating actors; presence of shared protocols across modules
Adaptability	Relatively low adaptability	High adaptability	Frequency of module modification; responsiveness to participant feedback; variation in program formats
Transition pathways	Fixed transitions between stages	Multiple transition and re-entry pathways	Transition completion rates; re-entry rates after temporary withdrawal; time between exit and re-entry
Technology role	Technology integrated uniformly across programs	Technology embedded as optional modular support	Optional technology uptake rates; continued use over time; user-reported usefulness or ease of use

This table provides a conceptual synthesis rather than empirically validated system categories. The listed dimensions are intended to clarify how unified and modular architectures may be distinguished and operationalized in future empirical research.

## 4 Technology as a modular component of recreational sport systems

The rapid expansion of digital technologies in sport has generated considerable interest in their potential to enhance learning, engagement, and feedback within recreational settings. Wearable sensors, mobile applications, and vision-based motion analysis tools are frequently promoted as accessible alternatives to laboratory-based systems. Despite these technical advances, many sport technologies fail to achieve sustained adoption or meaningful integration within recreational sport programs. From a system-level perspective, this limitation reflects not an inherent shortcoming of the technologies themselves, but a misalignment between technological solutions and the structural realities of recreational sport systems.

### 4.1 Technology adoption as a system-level challenge

Research on innovation diffusion and technology acceptance consistently demonstrates that adoption is shaped by social, organizational, and contextual conditions rather than by technical performance alone. Rogers' diffusion of innovations framework identifies relative advantage, compatibility, complexity, trialability, and observability as key determinants of whether innovations spread within social systems (11). Complementing this perspective, the Technology Acceptance Model emphasizes perceived usefulness and perceived ease of use as central predictors of user adoption (9).

In recreational sport environments, these adoption dynamics are intensified by participant heterogeneity and organizational constraints. Participants vary widely in technical literacy, motivation, and tolerance for complexity, while community sport organizations often rely on limited financial resources and volunteer-based staffing. Empirical research on digital sport technology adoption indicates that even when participants acknowledge potential benefits, concerns related to usability, contextual relevance, and disruption of established practices can impede sustained use (10).

These findings underscore that technology adoption in recreational sport cannot be examined in isolation from system design. Technologies introduced without explicit consideration of program structure, participant readiness, or pedagogical intent are likely to remain peripheral, inconsistently used, or ultimately abandoned.

### 4.2 Field-based sport technologies and scalability constraints

Reviews of consumer wearables and coaching technologies have documented persistent challenges associated with accuracy, interpretability, cost, and ecological validity when such tools are deployed beyond controlled laboratory settings (20–22). Although many technologies demonstrate acceptable validity under specific conditions, their scalability across heterogeneous recreational sport contexts remains constrained. Practical demands related to setup time, data processing, and interpretive expertise often exceed the operational capacity of community sport programs.

These limitations highlight the importance of distinguishing between technical feasibility and system compatibility. Field-based technologies may function reliably at a technical level yet remain systemically fragile if they impose requirements that conflict with existing program architectures. In recreational sport systems characterized by modular participation pathways and variable levels of engagement, technologies that assume uniform usage or sustained technical oversight may unintentionally exacerbate exclusion rather than promote access.

### 4.3 Technology as a modular system element

Adopting a system-oriented perspective reframes sport technology not as a universal solution, but as a modular component whose value depends on its alignment with participation stages and program design. Modular integration implies that technologies are selectively embedded within specific components of the system—such as introductory skill development, feedback-supported learning environments, or optional self-monitoring pathways—rather than imposed uniformly across all participants.

Design frameworks for sports interaction technologies reinforce this approach by emphasizing alignment between technological functions, user context, activity goals, and interaction constraints (22). Within recreational sport systems, such alignment requires that technologies support pedagogical objectives rather than redefine them. Feedback tools, for instance, may enhance learning at certain stages of participation while offering limited or even counterproductive value at others.

From a pedagogical perspective, motor learning research cautions against excessive reliance on augmented feedback, particularly when it undermines intrinsic motivation or attentional focus (17). Accordingly, modular technology integration must be guided not only by availability or novelty, but by learning principles and participant readiness.

### 4.4 Markerless motion capture as a case of modular integration

Recent developments in markerless motion capture and smartphone-based biomechanical analysis illustrate both the promise and the constraints of field-based sport technologies. Review and validation studies suggest that these systems can provide accessible kinematic information across a range of movements and sporting contexts (20, 21). Platforms such as OpenCap exemplify attempts to lower barriers to motion analysis by leveraging consumer hardware and automated processing workflows (23).

At the same time, methodological investigations reveal that accuracy, reliability, and interpretability remain highly context-dependent, with performance varying according to task characteristics, camera configurations, and user expertise (24). These findings support the argument that markerless motion capture technologies should not be framed as comprehensive replacements for traditional motion capture systems, nor as universally applicable solutions within recreational sport.

Instead, such technologies are best conceptualized as modular tools that may add value within specific system components—such as optional feedback modules, coach education contexts, or exploratory learning environments—when appropriately aligned with program objectives and user capabilities. Their effectiveness is shaped less by technical precision alone than by the manner in which they are embedded within the broader system architecture.

### 4.5 Implications for system design and innovation

Conceptualizing technology as a modular system component helps explain why many innovations struggle to scale in recreational sport environments. Technologies introduced without explicit consideration of participation stages, organizational capacity, or pedagogical coherence often remain isolated artifacts rather than integrated elements of the system. In contrast, modular integration allows technologies to be introduced incrementally, evaluated within context, and adapted over time.

This perspective aligns with broader recreational sport programming approaches that prioritize flexibility, inclusivity, and alignment between leadership, instruction, and participant experience. Technologies that reinforce these principles can contribute to system resilience, whereas those that conflict with them may intensify fragmentation.

Ultimately, the central challenge for recreational sport systems is not whether to adopt technology, but how to design architectures capable of accommodating innovation without undermining accessibility or sustainability. Addressing this challenge requires a shift away from technology-centered evaluation toward system-centered design.

**Figure 1:**
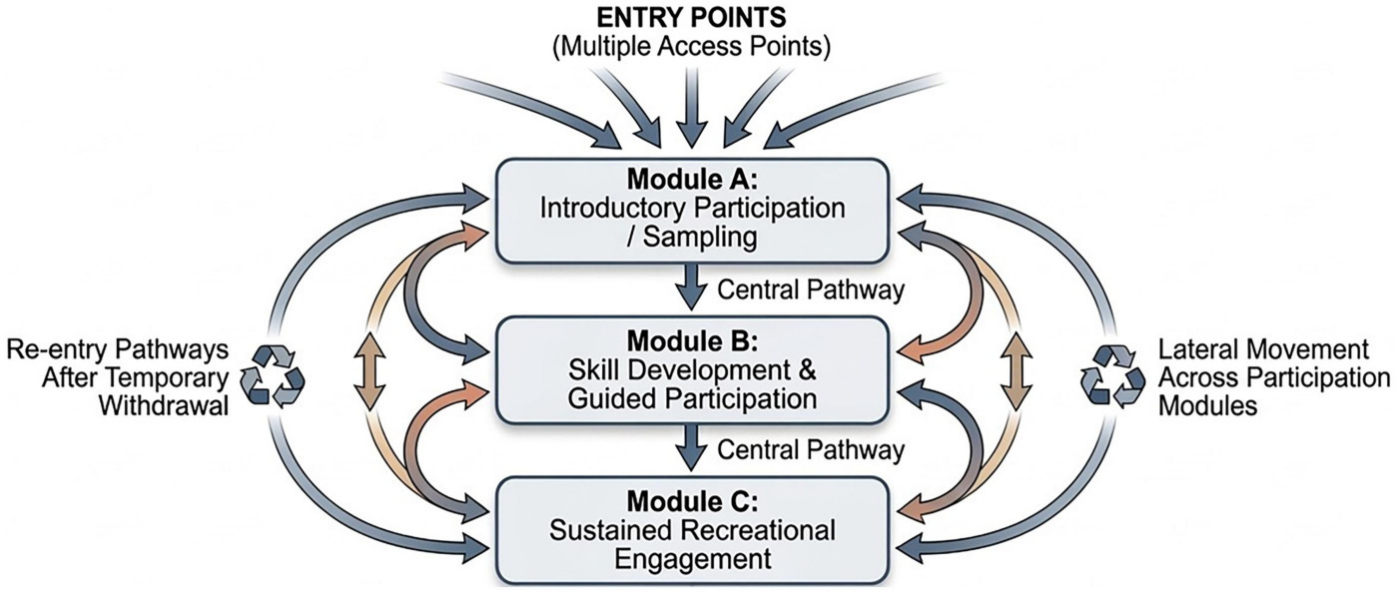
Conceptual illustration of a modular recreational sport system. The figure is not derived from empirical modelling or validated statistical relationships. It is intended as a heuristic representation of possible entry points, participation modules, lateral transitions, re-entry pathways, and optional technology-supported components. The illustrated relationships should be interpreted as conceptual relationships requiring future empirical testing rather than as demonstrated causal pathways. Optional technology nodes supporting participation modules: Coaching tools; Feedback systems; Self-monitoring technologies.

**Figure 2:**
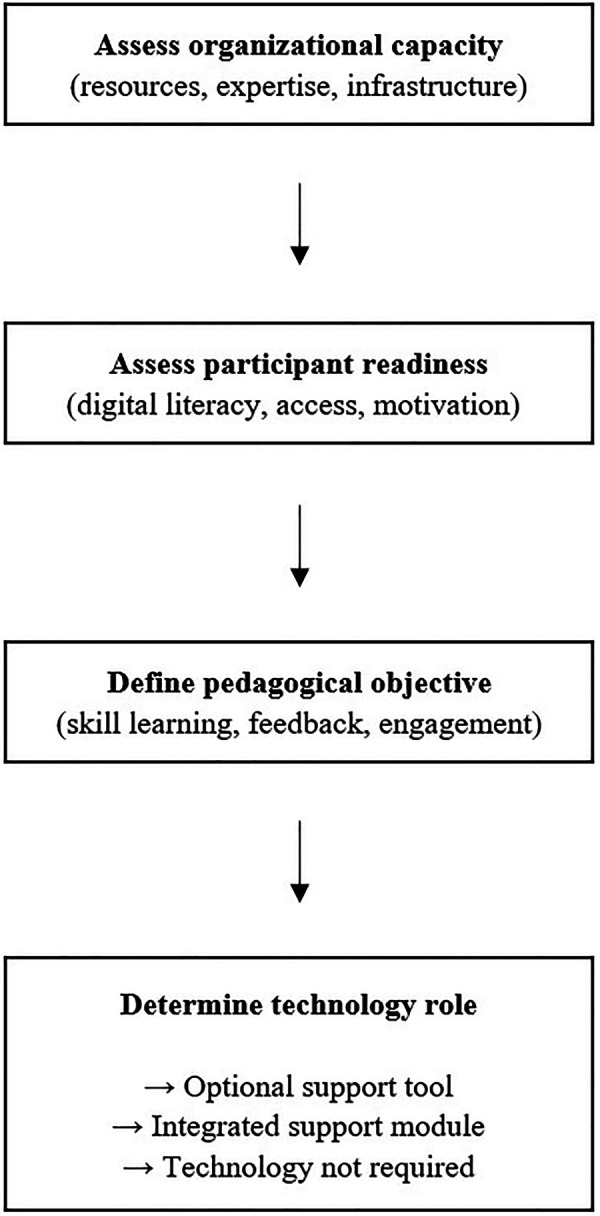
Conceptual decision aid for considering technology integration within recreational sport systems. This figure is intended as a heuristic tool rather than a validated decision model. It illustrates how organizational capacity, participant readiness, and pedagogical objectives may inform technology integration decisions. Future empirical work is needed to validate these decision pathways and identify measurable thresholds for each construct.

To illustrate the system architecture proposed in this paper, Figure 1 presents a conceptual illustration of a modular recreational sport system, highlighting participation modules, transition pathways, re-entry mechanisms, and optional technology-supported components. To complement this illustration, Figure 2 presents a conceptual decision aid illustrating how organizational capacity, participant readiness, and pedagogical objectives may inform the integration of technology within recreational sport systems.

## 5 Toward a conceptual framework for recreational sport system design

Recreational sport systems differ fundamentally from elite performance pathways in both purpose and structure. Rather than prioritizing the optimization of peak performance, recreational systems are oriented toward sustaining participation across diverse populations, contexts, and life stages. As a result, system design must emphasize accessibility, adaptability, and continuity in place of linear progression or early specialization. This section integrates participation theory, community sport research, and technology-enabled program design to articulate a conceptual framework for recreational sport system architecture.

### 5.1 Participation as a dynamic and non-linear process

Participation in recreational sport is best conceptualized as a dynamic and non-linear process shaped by interactions among individual, social, and environmental factors. Socioecological models of physical activity emphasize that participation decisions are not determined solely by individual motivation, but by the interplay of intrapersonal, interpersonal, organizational, and community-level constraints (19). This perspective challenges standardized program structures and highlights the need for systems capable of accommodating varied entry points, intensities, and participation trajectories.

Psychological models further reinforce this position. The Psychological Continuum Model (PCM) describes sport participation as movement through stages of awareness, attraction, attachment, and allegiance, with transitions that are neither uniform nor irreversible (18). In recreational contexts—where engagement is voluntary and situational—participants may shift between stages in response to life events, injury, or contextual barriers. Designing recreational sport systems therefore requires structural flexibility that supports re-entry, regression, and lateral movement without stigma or penalty.

These perspectives align with broader participation models integrating economic, behavioral, and sociological approaches. Syntheses of sport participation research demonstrate that sustained engagement is strongly influenced by perceived value, time availability, social support, and institutional accessibility (3). Collectively, this body of work underscores the limitations of rigid, linear participation models when applied to recreational sport environments.

### 5.2 Community sport programs as organizational systems

At the organizational level, recreational sport systems are typically situated within community-based, non-profit, or hybrid structures. Research on organizational capacity in community sport indicates that program sustainability depends on governance, human resources, partnerships, and adaptability rather than performance outcomes alone (2). From this perspective, recreational sport is more accurately understood as a system of coordinated programs than as a single unified pathway.

Program design literature in recreational sport management identifies modularity as a pragmatic response to participant diversity. Conceptual guides to recreational sport programming emphasize the value of offering varied formats, flexible scheduling, and multiple engagement intensities to accommodate differing motivations and constraints (25). Modular program structures enable participants to self-select experiences aligned with their goals—whether related to skill development, health, social interaction, or enjoyment—without requiring long-term specialization.

This modular orientation is consistent with system-level participation models that conceptualize recruitment, retention, and transition as interconnected yet distinct processes (1). In recreational contexts, transitions may occur not only between competitive levels but also across roles, such as from participant to volunteer or coach. Such dynamics further reinforce the need for systems capable of supporting multiple forms of engagement over time.

### 5.3 Inclusion, adaptation, and social objectives

A defining responsibility of recreational sport systems is to support inclusion across age, ability, and socio-cultural background. Sports-based youth development (SBYD) frameworks illustrate how sport programs can simultaneously promote physical activity, personal development, and social outcomes through intentional design (26). These models emphasize the integration of educational and psychosocial objectives alongside physical participation rather than treating them as secondary outcomes. Within the framework proposed in this paper, inclusivity is not treated as an auxiliary objective but as a structural design requirement of the participation system. Program modules, transition pathways, and technological components should therefore be designed to accommodate variation in ability, motivation, and access conditions.

Similarly, inclusive recreation literature stresses the importance of adapting environments, rules, and technologies to ensure access for individuals with disabilities or diverse functional capacities. Rather than framing inclusion as a specialized accommodation, inclusive recreation approaches position accessibility as a core system design principle. This orientation supports the argument that recreational sport systems should be adaptive by default, capable of accommodating diverse needs without segregating participants (27).

Empirical studies examining technology integration in recreational sport contexts further suggest that technological tools can support inclusion by lowering skill thresholds, offering individualized feedback, and enabling self-paced learning (12). However, such benefits are contingent on thoughtful system integration rather than isolated or technology-driven adoption.

### 5.4 Technology as a modular enabler, not a central driver

Within a system-oriented framework, technology should be understood as a modular enabler embedded within broader program architecture rather than as a central driver of participation. Field-based sport technologies—including video feedback systems, wearable sensors, and markerless motion capture—may enhance learning and engagement, but their impact depends on alignment with participant needs and organizational capacity (9, 11, 22).

Technology adoption research consistently demonstrates that perceived usefulness and perceived ease of use are necessary but insufficient conditions for sustained uptake; contextual fit and scalability are equally important (9, 11). In recreational sport systems characterized by wide variation in resources and technical expertise, technologies must remain optional, adaptable, and deployable at multiple levels of sophistication.

Evidence from sport education contexts supports this modular approach. Studies of video-based feedback in recreational and educational tennis environments indicate that even low-fidelity technologies can facilitate skill acquisition when embedded within pedagogically coherent programs (28). These findings suggest that the value of technology lies less in measurement precision than in its capacity to support reflection, autonomy, and engagement—central objectives of recreational sport.

### 5.5 Synthesis: principles for recreational sport system architecture

Synthesizing these perspectives, a conceptual framework for recreational sport system design can be articulated around four interrelated principles:

(1) Non-linearity: Participation pathways should enable flexible entry, exit, and re-engagement across life stages (18, 19).
(2) Modularity: Programs and technologies should operate as interchangeable components within a broader system rather than as a single unified pathway (1, 25).
(3) Inclusivity by design: Accessibility and adaptation should function as foundational system properties rather than supplementary features (26, 27).
(4) Context-sensitive technology integration: Technologies should enhance, rather than dictate, participation experiences and remain scalable across diverse community settings (12, 20, 21).

By framing recreational sport as a system rather than a pipeline, this framework redirects analytical attention from individual performance outcomes to the structural conditions that support long-term engagement. When positioned as a modular and optional component, technology can complement this architecture by enhancing feedback, learning, and motivation without constraining participation.

## 6 Discussion, implications, and future directions

### 6.1 Conceptual contributions

The central contribution of this paper lies in repositioning recreational sport as a system with its own architectural logic, rather than as a simplified extension of elite development pathways. Building on participation models that conceptualize recruitment, retention, and transition as interdependent processes, this analysis advances system-level thinking by situating recreational sport within a non-linear and modular framework (1, 6). In contrast to models that assume a single, unified progression, the proposed architecture explicitly recognizes multiple entry points, trajectories, and forms of engagement as legitimate system outcomes.

By integrating socioecological and psychological perspectives on participation, the framework extends earlier approaches that emphasized individual motivation or linear skill progression. Participation is instead conceptualized as an emergent outcome of interactions among personal goals, organizational structures, and contextual constraints. This shift foregrounds system resilience as a central design objective, highlighting the importance of architectures capable of accommodating fluctuation, temporary disengagement, and re-entry without systemic breakdown. In doing so, the framework redirects analytical attention from outcome optimization toward the structural conditions that may support participation continuity.

### 6.2 Technology repositioned within recreational sport systems

A key implication of this conceptual framework concerns the positioning of technology within recreational sport systems. Rather than treating digital tools as primary drivers of participation or performance, technology is reframed as a modular component embedded within broader system architecture. This positioning is consistent with diffusion of innovation and technology acceptance research, which demonstrates that adoption depends less on technical sophistication than on contextual compatibility, perceived usefulness, and organizational readiness (9, 11).

Evidence from field-based sport technology research reinforces this interpretation. Reviews of consumer wearables and coaching technologies consistently show that scalability, usability, and integration into existing practices are more consequential for impact than measurement precision alone (9–11). In recreational environments—where resources, expertise, and participant commitment vary widely—technologies that remain optional, adaptable, and context-sensitive are more likely to support sustained engagement. By conceptualizing technology as a subsystem rather than a central pillar, the framework avoids the common tendency toward technology-driven system design.

### 6.3 Implications for coaching and learning environments

The proposed system architecture also has important implications for coaching practice in recreational contexts. Recreational coaching environments prioritize enjoyment, autonomy, and long-term engagement rather than short-term performance optimization. Motor learning research consistently emphasizes the role of autonomy-supportive conditions and motivational climates in fostering intrinsic engagement and durable learning (17). When situated within a modular system, feedback technologies and instructional tools can be selectively deployed to support these principles by enabling individualized, self-paced learning rather than prescriptive correction.

Crucially, this framework does not advocate for the displacement of coach expertise by technology. Instead, coaches are positioned as system integrators who evaluate, adapt, and contextualize tools in relation to participant needs and program objectives. This role aligns with contemporary conceptualizations of coaching as a complex and adaptive practice operating within dynamic systems, rather than as a linear process of information transmission.

### 6.4 Implications for policy and community sport management

At the organizational and policy level, the framework offers an alternative basis for evaluating recreational sport programs beyond participation counts or competitive outcomes. Community sport organizations frequently face pressures to standardize provision in the interest of efficiency; however, research on organizational capacity indicates that adaptability and modularity are critical to sustainability in non-profit sport contexts (2). Conceptualizing programs as interconnected modules rather than fixed pathways enables organizations to respond more effectively to demographic diversity, fluctuating demand, and resource constraints.

This system-oriented perspective also aligns with broader sport development literature that prioritizes long-term engagement over early specialization or rigid progression models. Recognizing recreational participation as an outcome in its own right—not merely as a feeder to elite pathways—has implications for funding priorities, coach education frameworks, and evaluation criteria used by governing bodies and policymakers.

### 6.5 Future research directions

Although this paper advances a conceptual synthesis and does not empirically test the proposed framework, empirical research is required to examine how modular recreational sport systems function in practice. Future studies should investigate how different program configurations influence participation continuity, perceived inclusivity, and participant satisfaction across age groups and social contexts. Longitudinal designs would be particularly valuable for examining how individuals navigate between modules over time, thereby testing the non-linear participation assumptions underpinning the proposed framework.

Further research is also needed to examine technology integration at the system level rather than at the level of individual devices. Such work would clarify how digital tools can support scalable and inclusive recreational sport environments when embedded within coherent architectures. Consistent with the framework articulated here, future studies should prioritize ecological validity, organizational feasibility, and system-level outcomes.

Future empirical studies may also benefit from developing measurable system indicators capable of evaluating participation architecture. Potential indicators include re-entry rates following temporary withdrawal, transition smoothness between participation modules, participant retention across stages, and accessibility metrics reflecting the diversity of participants served by the system.

Future empirical studies could test the proposed conceptual propositions using longitudinal research designs that track participants across entry, transition, withdrawal, dropout, and re-entry points. Baseline analyses could examine differences in participant readiness, prior experience, motivation, and organizational capacity before participants enter modular or unified systems. Cluster analyses could identify distinct participation profiles, such as intermittent participants, lateral movers, sustained adopters, and re-entry participants. Because recreational sport participation often involves irregular attendance and temporary withdrawal, future studies should include explicit attrition handling strategies, missing-data procedures, and sensitivity analyses. Survival or time-to-event models may be useful for estimating the timing of dropout, re-entry, or sustained adoption and for testing whether specific modular system features are associated with longer participation continuity.

### 6.6 Concluding remarks

This paper presents a conceptual framework that positions recreational sport as a complex and adaptive system characterized by non-linearity, modularity, and inclusivity. By integrating participation theory, system design principles, and technology adoption research, the framework offers a coherent architecture for understanding and developing recreational sport environments. In shifting the analytical focus away from technology-centric or performance-centric narratives, this perspective emphasizes the structural conditions necessary to support sustained and meaningful participation across the lifespan.
